# Factors contributing to anaemia after uncomplicated falciparum malaria in under five year-old Nigerian children ten years following adoption of artemisinin-based combination therapies as first-line antimalarials

**DOI:** 10.1186/s12879-017-2876-9

**Published:** 2017-12-19

**Authors:** Akintunde Sowunmi, Bayo Fatunmbi, Kazeem Akano, Olubunmi A. Wewe, Chimere Agomo, Finomo Finomo, Joy Ebenebe, Nma Jiya, Jose Ambe, Robinson Wammanda, Godwin Ntadom, Olugbenga Mokuolu, George Emechebe, Nnenna Ezeigwe, Adejumoke I. Ayede, Elsie O. Adewoye, Grace O. Gbotosho, Onikepe A. Folarin, Christian T. Happi, Stephen Oguche, Wellington A. Oyibo, Francis Useh

**Affiliations:** 1 Antimalarial Therapeutic Efficacy Monitoring Group, National Malaria Elimination Programme, The Federal Ministry of Health, Abuja, Nigeria; 2 Department of Pharmacology and Therapeutics, University of Ibadan, Ibadan, Nigeria; 3 Institute for Medical Research and Training, University of Ibadan, Ibadan, Nigeria; 4 World Health Organization, Country Office, Kampala, Uganda; 5 Department of Medical Laboratory Science, University of Lagos, Lagos, Nigeria; 6 Department of Paediatrics, Federal Medical Centre, Yenagoa, Nigeria; 7 Department of Paediatrics, Nnamdi Azikiwe University, Awka, Nigeria; 8 Department of Paediatrics, Uthman Dan Fodio University, Sokoto, Nigeria; 9 Department of Paediatrics, University of Maiduguri, Maiduguri, Nigeria; 10 Department of Paediatrics, Ahmadu Bello University, Zaria, Nigeria; 11 Department of Paediatrics, University of Ilorin, Ilorin, Nigeria; 12 Department of Paediatrics, Imo State University Teaching Hospital, Orlu, Nigeria; 13 Department of Paediatrics, University of Ibadan, Ibadan, Nigeria; 14 Department of Physiology, University of Ibadan, Ibadan, Nigeria; 15 Department of Pharmacology and Toxicology, University of Ibadan, Ibadan, Nigeria; 16 Department of Biological Sciences and African Centre of Excellence for Genomics of Infectious Diseases (ACEGID), Redeemer University, Ede, Nigeria; 17 Department of Paediatrics, University of Jos, Jos, Nigeria; 18 Department of Medical Microbiology and Parasitology, University of Lagos, Lagos, Nigeria; 19 Department of Medical Laboratory Science, University of Calabar, Calabar, Nigeria; 20 Department of Clinical Pharmacology, University College Hospital, Ibadan, Nigeria

**Keywords:** *Plasmodium falciparum* malaria-associated anaemia, Artemisinin-based combination treatments, Children, Nigeria

## Abstract

**Background:**

Artemisinin-based combination therapies (ACTs) have remained efficacious treatments of acute falciparum malaria in many endemic areas but there is little evaluation of factors contributing to the anaemia of acute falciparum malaria following long term adoption of ACTs as first-line antimalarials in African children.

**Methods:**

Malarious <5 year-olds randomized to artemether-lumefantrine, artesunate-amodiaquine or dihydroartemisinin-piperaquine treatments were followed up clinically for 6 weeks. Anaemia was defined as haematocrit <30%; Malaria-attributable fall in haematocrit (MAFH) as the difference between haematocrit 28–42 days post- and pre-treatment; Total MAFH (TMAFH) as the difference between days 28–42 haematocrit and the lowest haematocrit recorded in the first week post-treatment initiation; Drug-attributable fall in haematocrit (DAFH) as the difference between MAFH and TMAFH; Early appearing anaemia (EAA) as haematocrit <30% occurring within 1 week in children with normal haematocrit pre-treatment. Predictors of anaemia pre-treatment, EAA, MAFH or DAFH >4% were evaluated by stepwise multiple logistic regression models. Survival analysis and kinetics of DAFH were evaluated by Kaplan-Meier estimator and non-compartment model, respectively.

**Results:**

Pre-treatment, 355 of 959 children were anaemic. Duration of illness >2 days and parasitaemia ≤10,000 μL^−1^ were independent predictors of anaemia pre-treatment. EAA occurred in 301 of 604 children. Predictors of EAA were age ≤ 15 months, history of fever pre-treatment and enrolment haematocrit ≤35%. The probabilities of progression from normal haematocrit to EAA were similar for all treatments. MAFH >4% occurred in 446 of 694 children; its predictors were anaemia pre-treatment, enrolment parasitaemia ≤50,000 μL^−1^, parasitaemia one day post-treatment initiation and gametocytaemia. DAFH >4% occurred in 334 of 719 children; its predictors were history of fever pre-and fever 1 day post-treatment initiation, haematocrit ≥37%, and parasitaemia >100,000 μL^−1^. In 432 children, declines in DAFH deficits were monoexponential with overall estimated half-time of 2.2d (95% CI 1.9–2.6). Area under curve of deficits in DAFH versus time and estimated half-time were significantly higher in non-anaemic children indicating greater loss of haematocrit in these children.

**Conclusion:**

After ten years of adoption of ACTs, anaemia is common pre-and early post-treatment, falls in haematocrit attributable to a single infection is high, and DAFH >4% is common and significantly lower in anaemic compared to non-anaemic Nigerian children.

**Trial registration:**

Pan African Clinical Trial Registry (PACTR) [PACTR201709002064150, 1 March 2017].

## Background

Artemisinin-based combination therapies (ACTs) have been adopted as first-line treatments of uncomplicated *Plasmodium falciparum* malaria by over 80 countries [1–3]. In many of these countries particularly those in southeast Asia, the period of adoption as first-line treatments has exceeded 10 years [4, 5]. In Nigeria, artemether-lumefantrine (AL) and artesunate-amodiaquine (AA) were adopted as first-line treatments in 2005 [6].

Anaemia, a consequence of treated and untreated falciparum malaria, is common before, during or after treatment in up to 40% of African children [7–9]. It is a major public health problem, and in anaemic patients, has been attributed to repeated infections within a short time frame, bone marrow dyserythropoiesis and increased splenic clearance of infected and non-infected erythrocytes [10–13]. Anaemia can also occur in African children with apparently asymptomatic infections [14, 15].

Despite adoption as first-line treatments in many countries for over a decade, there is little evaluation of the predictors of anaemia before, during or in the 1 week following initiation of treatment in malarious children. In malarious patients, the effects of infections and the drugs used to treat the infections can be evaluated using the following indices: malaria-attributable fall in haematocrit (MAFH), total malaria-attributable fall in haematocrit (TMAFH) [10], or drug-attributable fall in haematocrit (DAFH) [16]. Such evaluations are urgently needed as they can provide useful leads in the diagnosis and management of the anaemia following ACTs, and the extent to which adoption of ACTs as first-line antimalarials has modified the burden of the anaemia associated with malaria infections. From the clinical point of view, worsening of haematocrit levels or falls in haematocrit following ACTs of uncomplicated falciparum malaria in young African children, until proven otherwise, can be considered an adverse event attributable to the drug(s) used [17] – drug attributable falls in haematocrit [16]. However, DAFH can be attributed to interaction between the drug, the infection and the host.

For the reasons indicated above, the aims of present study were: using pre-determined cut-offs (haematocrit <30%, MAFH or DAFH >4%), to evaluate the predictors of the following: anaemia at presentation, progression from normal haematocrit at presentation to anaemia within 1 week of starting treatment (early-appearing anaemia [EAA]), and MAFH or DAFH >4% in a group of under 5 year old malarious children who were treated with AL, AA or dihydroartemisinin-piperaquine (DHP), during an open labelled therapeutic efficacy study. An additional aim was to evaluate the kinetics of the disposition of DAFH in anaemic and non-anaemic children following initiation of treatment.

## Methods

### Study locations

The study took place between June 2014 and September 2015. It was part of a programme to monitor antimalarial therapeutic efficacy at eight sentinel sites located in six geographical areas of Nigeria namely, Ogbia, Neni, Ogwa, Numan, Ilorin, Kura, Bodinga and Ibadan in Bayelsa, Anambra, Imo, Adamawa, Kwara, Kano, Sokoto and Oyo States, respectively. In virtually all study locations, malaria is hyperendemic and transmission occurs all year round; however, it is more intense during the rainy season from April to October. The details of the therapeutic efficacy study will be described elsewhere. The dataset presented here have not been previously published elsewhere.

### Study procedures

Standardized procedures and protocol were used at all sentinel sites. Briefly, patients were eligible to participate in the study if they were: aged 6–59 months, had symptoms compatible with acute uncomplicated malaria and *P. falciparum* mono-infections between 2000 and 200,000 μL^−1^ of blood, no history of antimalarial drug ingestion in the two weeks preceding enrolment, had no evidence of severe malaria [18, 19] and parents or guardians gave written informed consent.

Enrolled patients were randomized to AL, AA or DHP treatments for 3 days (day 0–2) as previously described [20]. The day of presentation (day of starting treatment) was regarded as day 0. Thick and thin blood films were obtained from each child as soon as they came to the clinic and the slides were carefully labelled with the patients’ codes and air-dried before being Giemsa-stained. Follow-up with clinical and parasitological evaluation was done daily on days 1–3 and 7, and thereafter, weekly for additional 5 weeks. Parasitaemia, asexual or sexual, in thick films was estimated by counting asexual and sexual parasites relative to 500 leukocytes, or 500 asexual or sexual forms whichever occurred first. From this figure, the parasite density was calculated assuming a leukocyte count of 6000 μL^−1^ of blood [21]. A slide was considered asexual parasite negative if no asexual parasite was detected after examination of 200 microscope fields. Parasite reduction ratio was defined as previously described [22].

### Haematological evaluation

Capillary blood obtained from a finger prick was collected before treatment and during follow-up, and was used to measure haematocrit using a microhaematocrit tube and microcentrifuge (Hawksley, Lancing, UK). Anaemia was defined as a haematocrit <30% and was further classified as mild, moderate or severe if haematocrit was 21–29%, 15–20% or <15%, respectively [8, 10].

### Definition of haematological parameters evaluated

**Fig. 1:**
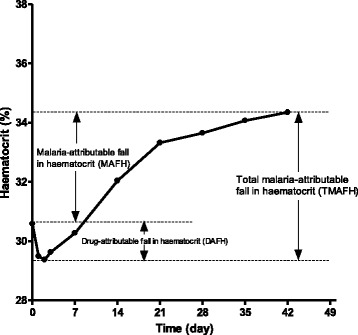
Parameters used for estimation of malaria-related falls in haematocrit (adapted from Price et al. [10] and Sowunmi et al. [16]

The following parameters described by Price et al. [10] and modified by Sowunmi et al. [16] were used for evaluation (Fig. 1):

(i) *Malaria-attributable fall in haematocrit (MAFH)* before treatment was defined as the difference between the patient’s maximum haematocrit value measured on days 28–42 and that on day 0 (pre-treatment).
(ii) *Total malaria-attributable fall in haematocrit (TMAFH)* was defined as the difference between the patient’s maximum haematocrit on days 28–42 and lowest haematocrit value recorded in the first week post-treatment initiation.
(iii) *Drug-attributable falls in haematocrit (DAFH)* was defined as the difference between MAFH and TMAFH, or as the difference between pre-treatment haematocrit and the lowest haematocrit recorded in the first week post-treatment initiation.
(iv) *Early-appearing anaemia (EAA)* was defined as haematocrit <30% occurring within 1 week in patients who were non-anaemic pre-treatment.
(v) *Anaemia recovery time:* Anaemia recovery time (in anaemic children pre-treatment) was defined as time elapsing from initiation of treatment to attainment of haematocrit value ≥30%. In non-anaemic children (pre-treatment) who subsequently progressed to anaemia following initiation of treatment (early-appearing anaemia), anaemia recovery time was defined as the time from the appearance of (onset of anaemia), to recovery from anaemia (until haematocrit rose to ≥30%).

### Kinetics of the disposition of drug-attributable falls in haematocrit

The kinetics of the deposition of the deficits in haematocrit from 30% using the lowest recorded haematocrit in the first week of initiating treatment (DAFH) were evaluated as previously described for the general evaluation of kinetics of the disposition of anaemia in malarious children [23, 24]. Briefly, in all children, haematocrit values below pre-treatment levels following initiation of treatment and at follow-up were subtracted from pre-treatment haematocrit at each time of measurement until haematocrit rose to or above pre-treatment level and the resulting values plotted against time. The final haematocrit when pre-treatment level was reached was therefore zero in all patients with falls in haematocrit following initiation of treatment. However, the final haematocrit at the time when pre-treatment haematocrit level was reached was assumed to be 0.01% [24]. The areas under the curve (AUC) of deficit in haematocrit from pre-treatment level versus time were obtained by trapezoidal rule using the computer program *Turbo Ken* (designed by Clinical Pharmacology Group, University of Southampton, United Kingdom) as previously described [23, 24]. AUC was also obtained manually by calculating the average haematocrit values between two consecutive time measurements and multiplying it by the time interval between the measurements, and summing up all the values, in a manner similar to that for the numerical estimation of area under a drug concentration-time curve (AUC) [25]. The unit of quantification would be %.d, if haematocrit values were used or g/L.d if haemoglobin values were used. Haematocrit values may be converted to haemoglobin values by dividing by 3 [26]. Semilogarithm plots of deficit in haematocrit from pre-treatment level versus time were plotted. The apparent terminal elimination rate constant (λ) was obtained by least-square regression analysis of the post-peak log-linear part of the plot of deficit in haematocrit from pre-treatment level versus time. The apparent terminal half-time of DAFH (t_1/2(DAFH)_) was calculated from ln 2/λ (that is, λt = 0.693). Only children in whom haematocrit values were measured at least 3 times following a drug-attributable fall in haematocrit post-initiation of treatment were included in this evaluation.

### Statistical analysis

Data were analyzed using version 6 of Epi-Info software [27] and the statistical program SPSS for Windows version 20.0 [28]. Variables considered in the analysis were related to the densities of *P. falciparum* asexual forms, haematocrit status at presentation and the values of DAFH and AUC_(DAFH)_. Proportions were compared by calculating χ^2^ using Yates’ correction, Fisher’s exact or Mantel Haenszel tests. Normally distributed, continuous data were compared by Student’s t test and analysis of variance (ANOVA). Kaplan-Meier estimator and pairwise log-rank tests were used to determine cumulative risk of anaemia occurring within 1 week post-treatment initiation with AL, AA or DHP. Stepwise multiple logistic regression models were used to test the association between: anaemia pre-treatment, normal haematocrit pre-treatment progressing to EAA within 1 week post-treatment initiation, malaria-attributable fall in haematocrit (MAFH) in excess of 4%, or drug-attributable fall in haematocrit (DAFH) in excess of 4%, and factors that were significant at univariate analysis. Correlation between the values of DAFH and AUC_(DAFH)_ in the same patients was assessed by Pearson’s correlation coefficient. Agreement between the values of DAFH and AUC_(DAFH)_ was assessed by Bland-Altman analysis [29]. Data were double entered serially using patients’ codes and were only analyzed at the end of the study.

## Results

### Characteristics of children enrolled in the study and parasite positivity following treatment

Nine hundred and ninety two children were enrolled and randomized to AL (*n* = 324), AA (*n* = 321) or DHP (*n* = 347) treatments. Of these children, haematocrit values were measured at presentation in 959 children (*n* = 315, 307 and 337 in AL, AA and DHP treatments, respectively). Mean weight of 959 children enrolled in the study was 13.2 kg (95% CI 12.9–13.4). The other characteristics of these children at presentation are shown in Table 1. Two hundred and fifty six of the 959 children (27%) were aged ≤24 months and 121 (13%) were aged ≤15 months. One hundred and one (11%) had asexual parasitaemia >100,000 μL^−1^. Overall, mean haematocrit at presentation was 30.6% (95% CI 30.3–30.9). At presentation, anaemia was present in 355 of 959 children (37%) and it was mild, moderate or severe in 323 (33.7%), 30 (3.1%) or 2 children (0.2%), respectively (Table 1). Overall, asexual parasite positivity on day 3 was 29 of 959 children (3%) and it was similar in all treatments groups [11 of 315 children (3%), 12 of 307 children (4%), and 6 of 337 children (2%) in AL-, AA- and DHP-treated children, respectively, *P* = 0.24].

**Table 1:** Characteristics of the 959 children enrolled in the study of risk factors for anaemia in uncomplicated falciparum malaria

Variables	No. with enrolment haematocrit, n (%)^a^	Enrolment haematocrit, mean [95% CI]	Anaemia (haematocrit <30%) at presentation, n (%)^a^			
			ALL	Mild	Moderate	Severe
Total	959 (96.7)	30.6 [30.3–30.9]	355 (37)	323 (33.7)	30 (3.1)	2 (0.2)
Male	522 (52.6)	30.5 [30.1–31]	197 (20.5)	182 (19)	14 (1.5)	1 (0.1)
Female	437 (44.1)	30.7 [30.2–31.2]	158 (16.5)	141 (14.7)	16 (1.6)	1 (0.1)
Aged^b^ ≤ 15 months	121 (12.6)	29 [28.1–30]	56 (5.8)	48 (5)	8 (0.8)	0 (0)
≤ 24 months	256 (25.8)	29.7 [29.1–30.3]	108 (11.3)	93 (9.7)	14 (1.5)	1 (0.1)
> 24 months	695 (70.1)	30.9 [30.6–31.2]	244 (25.4)	228 (23.8)	15 (1.6)	1 (0.1)
Asexual parasitaemia^c^						
≤ 25,000 μL^−1^	559 (52.3)	30.6 [29.6–30.4]	225 (23.5)	203 (21.2)	20 (2.1)	2 (0.2)
> 25,000–50,000 μL^−1^	141 (14.7)	31.2 [30.3–32.2]	41 (4.3)	35 (3.6)	6 (0.6)	0 (0)
> 50,000–100,000 μL^−1^	152 (15.8)	31.3 [30.4–32.1]	52 (5.4)	50 (5.2)	2 (0.2)	0 (0)
> 100,000 μL^−1^	101 (10.5)	31.4 [30.4–32.4]	35 (3.6)	33 (3.4)	2 (0.2)	0 (0)
Gametocytaemia						
Present	47 (4.7)	28.8 [27.6–30]	22 (2.3)	20 (2.1)	2 (0.2)	0 (0)
Absent	912 (91.9)	30.7 [30.3–31]	333 (34.7)	303 (31.6)	28 (2.9)	2 (0.2)
Region of enrolment						
Eastern flank^d^	503 (50.7)	30.6 [30.1–31.1]	175 (18.2)	152 (15.8)	21 (2.2)	2 (0.2)
Western flank^e^	456 (46)	30.6 [30.1–31]	180 (18.8)	171 (17.8)	9 (0.9)	0 (0)

^a^values in parenthesis are expressed as percentage of 992 children randomized

^b^data for age not available in eight children

^c^data for asexual parasitaemia not available in 6 children

^d^Eastern flank includes: Numan, Nena, Ogbia and Ogwa in Adamawa, Anambra, Bayelsa and Imo States, respectively

^e^Western flank includes: Kura, Ilorin, Ibadan and Bodinga in Kano, Kwara, Oyo and Sokoto States, respectively

### Factors contributing to anaemia at presentation

Factors associated with anaemia at presentation are presented in Table 2. In a univariate analysis, an age ≤ 15 months, duration of illness >2 days, and enrolment asexual parasitaemia ≤10,000 μL^−1^ were associated with anaemia at presentation. In a multivariate analysis, duration of illness >2 days and an enrolment asexual parasitaemia ≤10,000 μL^−1^ were independent predictors of anaemia at presentation (Table 2).

**Table 2:** Predictors of anaemia at presentation in children <5 years with uncomplicated falciparum malaria

Variable	Total no.	No. with anaemia at presentation	OR (95% CI)	*P* value	AOR (95% CI)	*P* value
Gender						
Female	437	158	1			
Male	522	197	1.1 (0.8–1.4)	0.66	–	–
Age (months)						
> 15	837	296	1		1	
≤ 15	114	56	1.8 (1.2–2.6)	0.01	1.5 (0.9–2.5)	0.09
Duration of illness (days)						
≤ 2	295	92	1		1	
> 2	330	138	1.6 (1.1–2.2)	0.01	1.5 (1.1–2.1)	0.02
Enrolment body temperature (°C)						
≤ 37.4	357	143	1			
> 37.4	601	211	0.8 (0.6–1.1)	0.14	–	–
History of fever						
Absent	169	70	1			
Present	789	284	1.2 (0.9–1.8)	0.22	–	–
Asexual parasitaemia (μL^−1^)						
> 10,000	389	160	1		1	
≤ 10,000	564	193	1.3 (1.1–1.8)	0.003	1.5 (1.1–2.2)	0.02
Gametocyte carriage at presentation						
Absent	912	333	1			
Present	47	22	1.5 (0.6–2.8)	0.2	–	–
Season of enrolment						
Dry (November–March)	69	24	1			
Wet (April–October)	890	331	1.1 (0.7–1.9)	0.79	–	–
Region of enrolment						
Eastern flank^a^	456	180	1			
Western flank^b^	503	175	0.8 (0.6–1.1)	0.15	–	–

*OR* odd ratio, *AOR* adjusted odd ratio, *CI* confidence interval

^a^Eastern flank includes: Numan, Neni, Ogbia and Ogwa in Adamawa, Anambra, Bayelsa and Imo States, respectively

^b^Western flank includes: Kura, Ilorin, Ibadan and Bodinga in Kano, Kwara, Oyo and Sokoto States, respectively

### Recovery from pre-treatment anaemia

Data for the evaluation of recovery from pre-treatment anaemia were available in 318 of 355 children. Of these children, 142, 101, 58, 26, 8 and 3 children recovered from their anaemia within 1, 2, 3, 4, 5 and 6 weeks post-treatment initiation, respectively. Overall, mean anaemia recovery time was 11.5 days (95% CI 10.5–12.5, *n* = 308). Mean anaemia recovery time was significantly longer in DHP-treated children compared with AL- and AA-treated children [13.3 days (95% CI 11.5–15.2, *n* = 95) versus 11.7 days (95% CI 9.9–13.6, *n* = 108) versus 9.5 days (95% CI 8–11, *n* = 105) respectively, *P* = 0.01]. Ten children did not recover from their pre-treatment anaemia during the entire follow-up period.

### Factors contributing to progression from normal haematocrit pre-treatment to anaemia within 1 week post –treatment initiation (early-appearing anaemia)

**Fig. 2:**
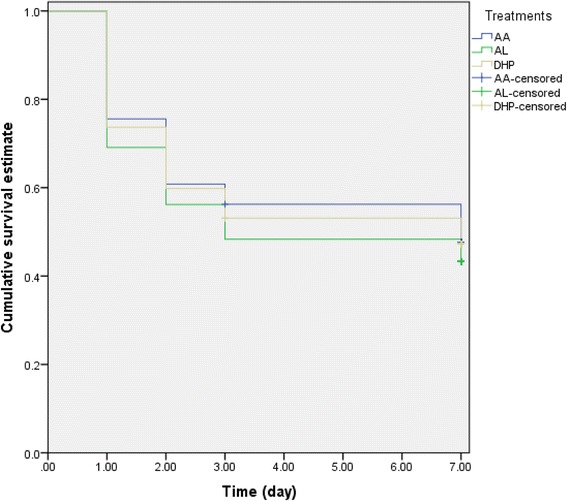
Kaplan-Meier survival estimates of anaemia occurring within 1 week following treatment with artesunate-amodiaquine (AA, blue line), artemether-lumefantrine (AL, green line) or dihydroartemisinin-piperaquine (DHP, yellow line) in children who were non-anaemic pre-treatment. Log-rank statistic = 1.24, *P* = 0.54. Pooled data from all sentinel sites. The probabilities of progression to anaemia within 1 week post-treatment were similar with all three treatments

Data for evaluation progression from normal haematocrit pre-treatment to an early-appearing anaemia (EAA) within 1 week post-treatment initiation were available in 604 children. Of these children, 301 children (50%) progressed to anaemia within 1 week post-treatment initiation. The proportions of non-anaemic children who subsequently progressed to anaemia within 1 week of starting treatment were similar with all three treatments (101 of 191 (53%) versus 90 of 187 (48%) versus 110 of 226 children (49%) in AL, AA and DHP treatments, respectively *P* = 0.59). The probabilities of progression to anaemia 1 week post-treatment initiation were also similar in all three treatments (Log-rank statistic = 1.24; *P* = 0.54, Fig. 2). Factors contributing to progression to anaemia within 1 week are shown in Table 3. In a univariate analysis, an age ≤ 15 months, history of fever at presentation, enrolment haematocrit ≤35% and persistent asexual parasitaemia till 1 or 2 days post-treatment initiation were significantly associated with progression to anaemia within 1 week post-initiation of treatment. In a multivariate analysis, an age ≤ 15 months, history of fever at presentation and enrolment haematocrit ≤35% were independent predictors of progression to anaemia within 1 week (Table 3).

**Table 3:** Predictors of progression from normal haematocrit pre-treatment to anaemia within one week post-initiation of artemisinin-based combination treatments in <5 year-old children with uncomplicated falciparum malaria

Variable	Total no.	No. with anaemia 1 week post-initiation of ACTs	OR (95% CI)	P value	AOR (95% CI)	*P* value
Gender						
Female	278	132	1			
Male	325	168	0.8 (0.6–1.2)	0.34	–	–
Age (month)						
> 15	541	255	1		1	
≤ 15	58	42	2.9 (1.6–5.4)	< 0.0001	2.5 (1.4–4.7)	0.003
Duration of illness (day)						
≤ 2	203	101	1			
> 2	192	100	1.0 (0.7–1.6)	1.0	–	–
Enrolment body temperature (°C)						
≤ 37.4	214	104	1			
> 37.4	390	197	1.1 (1.0–1.9)	0.71	–	–
History of fever at enrolment						
Absent	99	39	1		1	
Present	505	262	1.7 (1.1–2.6)	0.03	1.8 (1.1–2.9)	0.01
Fever on day 1						
Absent	501	255	1			
Present	70	38	1.1 (0.7–1.9)	0.69	–	–
Haematocrit (%)						
> 35	140	42	1		1	
≤ 35	464	259	2.9 (2.0–4.4)	< 0.0001	2.9 (1.9–4.4)	<0.0001
Parasitaemia (μL^−1^)						
≤ 50,000	434	213	1			
> 50,000	166	87	1.1 (0.8–1.6)	0.52	–	–
Parasitaemia on day 1						
Absent	244	111	1			
Present	360	190	1.3 (1.0–1.9)	0.09	–	–
Parasitaemia day 1 or 2						
Absent	233	104	1		1	
Present	371	197	1.4 (1.0–2.0)	0.04	1.3 (0.9–1.9)	0.11
Gametocytaemia						
Absent	579	289	1			
Present	25	12	0.9 (0.4–2.1)	1.0	–	–
Parasite clearance time (day)						
≤ 2	468	234	1			
> 2	136	67	1.0 (0.7–1.4)	0.96	–	–
Fever clearance time (day)						
≤ 2	367	186	1			
> 2	13	9	1.9 (0.9–4.3)	0.13	–	–
Season of enrolment						
Dry (November–March)	45	17	1			
Wet (April–October)	559	284	1.7 (0.9–3.2)	0.13	–	–
Drug treatment						
DHP	226	110	1			
AL	191	101	1.2 (0.8–1.7)	0.49	–	–
AA	187	90	1.0 (0.7–1.4)	1.0		

*OR* odd ratio, *AOR* adjusted odd ratio, *CI* confidence interval, *DHP* dihydroartemisinin-piperaquine, *AL* artemether-lumefantrine, *AA* artesunate-amodiaquine, *ACTs* artemisinin-based combination treatments

### Recovery from early-appearing anaemia

Data for evaluation of recovery from EAA were available in 275 of 301 children. Of these children, 166, 71, 19, 9, 3 and 2 children recovered at 1, 2, 3, 4, 5 and 6 weeks post-treatment initiation, respectively. Overall, mean anaemia recovery time was 8.4 days (95% CI 7.4–9.3, *n* = 270). Anaemia recovery times were similar with all three treatments [8.6 days (95% CI 6.8–10.4, *n* = 91) versus 7.3 days (95% CI 6–8.5, *n* = 80) versus 9.5 days (95% CI 8–11, *n* = 99) in AL-, AA- and DHP-treated children, respectively *P* = 0.14]. Five children did not recover from their anaemia during the entire follow-up period. Anaemia recovery time in children who progressed to EAA was significantly shorter than anaemia recovery time of children who were anaemic at presentation [8.4 days (95% CI 7.4–9.3, *n* = 270) versus 11.5 days (95% CI 10.5–12.5, *n* = 308), respectively *P* < 0.0001].

### Time-course of haematocrit following artemisinin-based combination treatments

**Fig. 3:**
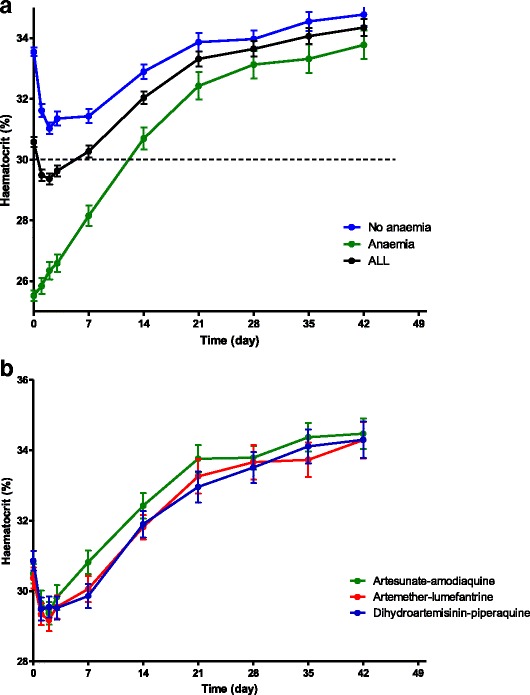
Time-course of haematocrit in (**a**) all (black line), anaemic (green line) and non-anaemic (blue line) children following artemisinin-based combination treatments, and (**b**) in children treated with artemether-lumefantrine (red line), artesunate-amodiaquine (green line) or dihydroartemisinin-piperaquine (blue line). Values represent means of haematocrit and standard error of means. Note little or no fall in haematocrit in anaemic children following treatment

Figure 3a shows the time-course of haematocrit in children who were non-anaemic at presentation and those who were anaemic at presentation following ACTs. Following treatment, there was little or no fall in haematocrit in anaemic children compared to non-anaemic children indicating little or no drug-attributable falls in the former. Additionally, the rate of rise in haematocrit was rather ‘steep’ in anaemic children following treatment. Following initiation of treatment, overall, mean time to nadir hematocrit was reached 2.2 days (95% CI 2.1–2.4, *n* = 719) after initiation of treatment and it was significantly shorter in anaemic compared to non-anaemic children at presentation [1.4 days (95% CI 1.3–1.7, *n* = 213) versus 2.6 days (95% CI 2.5–2.8, *n* = 506) in anaemic and non-anaemic children, respectively, *P* < 0.0001]. Compared with pre-treatment haematocrit, falls in haematocrit to nadir levels were significantly lower in anaemic compared to non-anaemic children [3.4% (95% CI 3.1–3.8 *n* = 213) versus 5.6% (95% CI 5.3–6, *n* = 506, respectively, *P* < 0.0001]. When falls to nadir haematocrits were expressed as percentages of pre-treatment haematocrit levels, falls were significantly higher in non-anaemic compared to anaemic children [16.3% (95% CI 15.5–17.2, *n* = 506) versus 13.1% (95% CI 11.9–14.4, *n* = 213), respectively *P* < 0.0001]. The time-course of haematocrit were similar with all three treatments (Fig. 3b). Mean time to nadir haematocrit was also similar with all three treatments [2.1 days (95% CI16.8–2.3, *n* = 234) versus 2.2 days (95% CI 2–2.4, *n* = 234) versus 2.4 days (95% CI 2.1–2.6, *n* = 251) in AL-, AA- and DHP-treated children, respectively *P* = 0.24]. Similarly, when falls to nadir haematocrits were expressed as percentages of pre-treatment haematocrit levels, falls were not significantly different between all three treatments [15.3% (95% CI 14.1–16.6, *n* = 234) versus 14.8% (95% CI 13.6–16.1, *n* = 234) versus 16% (95% CI 14.7–17.2, *n* = 251) in AL-, AA- and DHP-treated children, respectively *P* = 0.44].

### Malaria-attributable fall in haematocrit

Data for estimation of MAFH were available in 748 children (428 and 320 non-anemic and anaemic children, respectively). In these children, there was no malaria-attributable fall in haematocrit following infection in 54 children (49 and 5 non-anaemic and anaemic children, respectively). The proportion of children with no MAFH following treatment was significantly higher in non-anaemic children compared with anaemic children at presentation (*P* < 0.0001). Varying degrees of malaria-attributable falls in haematocrit were seen in 694 children (379 and 315 in non-anaemic and anaemic children, respectively). Overall, mean MAFH was 6.8% (95% CI 6.5–7.2, *n* = 694) and it was significantly lower in non-anemic compared to anaemic children [4.6% (95% CI 4.3–4.9, *n* = 379) versus 9.5% (95% CI 9–10, *n* = 315), respectively *P* < 0.0001)].

MAFH in excess of 4% occurred in 446 of 694 children (64.3%). The proportion of non-anaemic children with MAFH in excess of 4% was significantly lower compared to anaemic children (170 of 379 children (44.9%) versus 276 of 315 children (87.6%), respectively; *P* < 0.0001) indicating a greater burden of a single episode of infection was borne by anaemic compared to non-anaemic children. Mean MAFH in non-anaemic children were similar for all three treatments [4.8% (95% CI 4.3–5.3, *n* = 117) versus 4.8% (95% CI 4.3–5.3, *n* = 127) versus 4.8% (95% CI 4.3–5.3, *n* = 135) in AL, AA and DHP- treated children, respectively, *P* = 0.33]. Similarly, mean MAFH in anaemic children were similar for all three treatments [9.4% (95% CI 8.6–10.2, *n* = 112) versus 9.8% (95% CI 8.9–10.6, *n* = 107) versus 9.3% (95% CI 8.3–10.3, *n* = 96) in AL, AA and DHP -treated children, respectively, *P* = 0.75]. The proportions of children with MAFH in excess of 4% were also similar for all three treatments in non-anaemic [56 of 117 versus 58 of 127 versus 56 of 135 children in AL. AA and DHP -treated children, respectively, *P* = 0.58] and anaemic children [98 of 112 versus 97 of 107 versus 81 of 96 children in AL-, AA- and DHP-treated children, respectively, *P* = 0.4].

Factors associated with MAFH in excess of 4% are shown in Table 4. In a univariate analysis, an age ≤ 38 months, anaemia at presentation, enrolment parasitaemia ≤50,000 μL^−1^, parasitaemia 1 day after start of treatment and gametocytaemia within 1 week were associated with MAFH >4%. In a multivariate analysis, anaemia at presentation, enrolment parasitaemia ≤50,000 μL^−1^, parasitaemia 1 day after start of treatments and gametocytaemia within 1 week were independent predictors of MAFH >4% (Table 4).

**Table 4:** Predictors of malaria-attributable fall in haematocrit (MAFH) in excess of 4% in <5 year-old children with uncomplicated falciparum malaria following artemisinin-based combination treatments

Variable	Total no.	No. with MAFH >4%	OR (95% CI)	*P* value	AOR (95% CI)	*P* value
Gender						
Female	314	203	1			
Male	380	243	1.0 (0.7–1.3)	0.91	–	–
Age (month)						
> 38	354	213	1		1	
≤ 38	335	228	1.4 (1.0–1.9)	0.04	2.1 (0.8–1.6)	0.53
Duration of illness (day)						
≤ 2	200	120	1			
> 2	274	180	1.1 (0.7–1.6)	0.78	–	–
Enrolment body temperature (°C)						
≤ 37.4	264	173	1			
> 37.4	429	272	0.9 (0.7–1.3)	0.63	–	–
History of fever at enrolment						
Absent	123	77	1			
Present	571	368	1.1 (0.7–1.6)	0.78	–	–
Fever on day 1						
Absent	619	398	1			
Present	62	38	0.9 (0.5–1.5)	0.74	–	–
Haematocrit (%)						
≥ 30	379	170	1		1	
< 30	315	276	8.7 (5.9–12.9)	< 0.0001	9.4 (6.3–14.1)	<0.0001
Parasitaemia (μL^−1^)						
> 50,000	171	91	1		1	
≤ 50,000	521	355	1.9 (1.3–2.7)	0.001	1.9 (1.3–2.9	0.002
Parasitaemia on day 1						
Absent	380	228	1		1	
Present	314	218	1.5 (1.1–2.1)	0.01	1.6 (1.1–2.3)	0.01
Gametocytaemia (D0–7)						
Absent	621	390	1		1	
Present	73	56	2.0 (1.1–3.4)	0.027	2.0 (1.0–3.7)	0.04
Parasite clearance time (days)						
≤ 2	551	357	1			
> 2	142	89	0.9 (0.6–1.3)	0.71	–	–
Fever clearance time (days)						
≤ 2	416	261	1			
> 2	13	11	3.3 (0.7–14.9)	0.19	–	–
Season of enrolment						
Dry (November–March)	41	23	1			
Wet (April–October)	653	423	1.4 (0.8–2.7)	0.34	–	–
Drug treatment						
DHP	231	137	1			
AL	229	154	1.4 (1.0–2.1)	0.1	–	–
AA	234	155	1.3 (0.9–2.0)	0.15		

*OR* odd ratio, *AOR* adjusted odd ratio, *CI* confidence interval, *MAFH* malaria-attributable fall in haematocrit, *DHP* dihydroartemisinin-piperaquine, *AL* artemether-lumefantrine, *AA* artesunate-amodiaquine. D0–7, day 0–7

### Drug-attributable falls in haematocrit

**Fig. 4:**
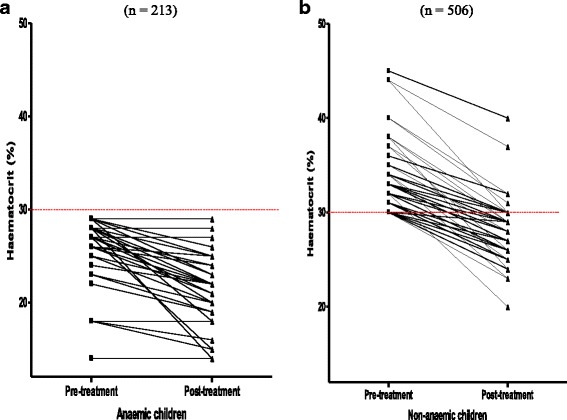
Drug-attributable falls in haematocrit in anaemic **(a)** and non-anaemic children **(b)** following artemisinin-based combination treatments

Data for estimation of DAFH were available in 929 children (588 and 341 non-anaemic and anaemic children at presentation, respectively). In these children, there was no DAFH following treatment in 210 children (82 and 128 non-anaemic and anaemic children, respectively). The proportions of children without DAFH were significantly higher in anaemic compared to non-anaemic children (*P* < 0.0001, Fig. 4). Varying degrees of DAFH occurred in 719 children (506 and 213 non-anaemic and anaemic children, respectively; Fig. 4). Overall, lowest falls from pre-treatment levels were seen on day 1, 2 or 3 in 232, 217 or 158 children, respectively. Overall, mean DAFH was 5% (95% CI 4.7–5.2, *n* = 719) and it was significantly higher in non-anaemic compared to anaemic children [5.6% (95% CI 5.3–5.9, *n* = 506) versus 3.4% (95% CI 3.1–3.8, *n* = 213), *P* < 0.0001; Fig. 4) [see also time-course of changes in haematocrit following treatments above]. DAFH in excess of 4% occurred in 385 of 719 children (53.5%) [279 of 506 (55.1%) and 55 of 213 (25.8%) in non-anaemic and anaemic children, respectively]. The proportion of non-anaemic children with DAFH in excess of 4% was significantly higher than in anaemic children (*P* < 0.0001) indicating relative lack of conservation in non-anaemic children and relative conservation in anaemic children.

DAFH in non-anaemic children were similar for all three treatments [5.7% (95% CI 5.1–6.2, *n* = 167) versus 5.5% (95% CI 4.9–6.1, *n* = 155) versus 5.6% (95% CI 5.1–6.2, *n* = 184) in AL-, AA- and DHP-treated children, respectively, *P* = 0.93]. Similarly, mean DAFH in anaemic children were similar for all three treatments [3.2% (95% CI 2.7–3.7, *n* = 67) versus 3.2% (95% CI 2.7–3.8, *n* = 79) versus 3.9% (95% CI 3.2–4.5, *n* = 67) in AL-, AA- and-treated children, respectively, *P* = 0.2]. The proportions of children with DAFH in excess of 4% were also similar for all three treatments in non-anaemic [97 of 167 (58%) versus 82 of 155 (53%) versus 100 of 184 children (54%) in AL-, AA- and DHP-treated children, respectively *P* = 0.62] and in anaemic children [16 of 67 (24%) versus 19 of 79 (24%) versus 20 of 67 children (30%) in AL-, AA- and DHP-treated children, respectively *P* = 0.66].

Factors associated with DAFH in excess of 4% are shown in Table 5. In a univariate analysis, enrolment body temperature ≥ 38°C, history of fever at presentation, fever 1 day post-treatment initiation, enrolment haematocrit ≥37%, enrolment parasitaemia >100,000 μL^−1^, no gametocytaemia at presentation and within one week post-treatment initiation were significantly associated with DAFH >4%. In a multivariate analysis, history of fever at presentation, fever 1 day post-treatment initiation, enrolment haematocrit ≥37%, and enrolment parasitaemia >100,000 μL^−1^ were independent predictors of DAFH >4% (Table 5).

**Table 5:** Predictors of drug-attributable falls in haematocrit (DAFH) in excess of 4% in <5 year-old children with uncomplicated falciparum malaria following artemisinin-based combination treatments

Variable	Total no.	No. with DAFH >4%	OR (95% CI)	*P* value	AOR (95% CI)	*P* value
Gender						
Female	323	152	1			
Male	395	181	0.8 (0.6–1.1)	0.15	–	–
Age (month)						
> 15	627	291	1			
≤ 15	88	40	1.0 (0.6–1.5)	0.96	–	–
Duration of illness (day)						
≤ 2	235	125	1			
> 2	262	122	0.8 (0.5–1.1)	0.17	–	–
Enrolment body temperature (°C)						
< 38	389	159	1		1	
≥ 38	330	175	1.6 (1.2–2.2)	0.001	1.3 (1.0–1.8)	0.09
History of fever at enrolment						
Absent	109	26	1		1	
Present	610	308	3.3 (2.0–5.2)	< 0.0001	3.0 (1.8–4.9)	<0.0001
Fever on day 1						
Absent	611	267	1		1	
Present	91	58	2.3 (1.4–3.6)	0.001	1.8 (1.1–2.9)	0.02
Haematocrit (%)						
< 37	641	271	1		1	
≥ 37	78	63	5.7 (3.2–10.3)	< 0.0001	5.5 (3.0–10.2)	<0.0001
Parasitaemia (μL^−1^)						
≤ 100,000	631	227	1		1	
> 100,000	85	55	2.3 (1.5–3.8)	< 0.0001	2.1 (1.3–3.5)	0.004
Parasitaemia on day 1						
Absent	288	133	1			
Present	431	201	1.0 (0.8–1.4)	0.84	–	–
Gametocytaemia (D0)						
Present	31	7	1		1	
Absent	688	327	3.1 (1.3–7.3)	0.01	1.7 (0.5–5.8)	0.36
Gametocyteamia (within D0–7)						
Present	61	16	1		1	
Absent	658	318	2.6 (1.5–4.7)	0.001	1.4 (0.6–3.3)	0.44
Parasite clearance time (day)						
≤ 2	563	259	1			
> 2	155	75	1.1 (0.8–1.6)	0.66	–	–
Fever clearance time (day)						
≤ 2	455	223	1			
> 2	19	10	1.2 (0.5–2.9)	0.94	–	–
Season of enrolment						
Dry (November–March)	43	16	1			
Wet (April–October)	676	318	1.5 (0.8–2.8)	0.27	–	–
Drug treatment						
DHP	251	120	1			
AL	234	113	1.0 (0.7–1.5)	0.99	–	–
AA	234	101	0.8 (0.6–1.2)	0.35		

*OR* odd ratio; *AOR* adjusted odd ratio, *CI* confidence interval, *DAFH* drug-attributable fall in haematocrit, *DHP* dihydroartemisinin-piperaquine, *AL* artemether-lumefantrine, *AA* artesunate-amodiaquine

Of the non-anaemic children who subsequently became anaemic 1 week after start of treatment [EAA], 245 of 298 children (82%) had DAFH >4%. At a DAFH of 4%, 84 of 301 non-anaemic children at presentation (28%) progressed to anaemia within 1 week. At a DAFH of 10%, 121 of 301 non-anaemic children at presentation (40%) progressed to anaemia within 1 week.

### Kinetics of the disposition of drug-attributable falls in haematocrit

**Fig. 5:**
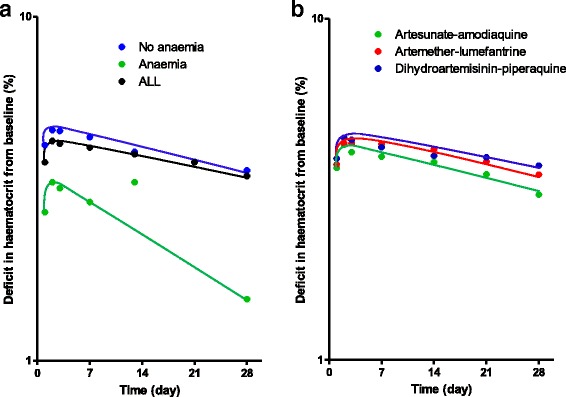
Semilog plots of deficit in haematocrit from baseline versus time (**a**) in all children (black line), anaemic (green line) and non-anaemic children (blue line) and (**b**) in children treated with artemether lumefantrine (red line), artesunate-amodiaquine (green line) or dihydroartemisinin-piperaquine (blue line)

The demographic and other characteristics of the 432 children enrolled in kinetics of the disposition of DAFH study (*n* = 148 for AL, *n* = 138 for AA and *n* = 146 for DHP) are shown in Table 6. Overall, there was a monoexponential decline of the deficits in DAFH with an estimated mean half-time (t_½el_) of 2.2 days (95% CI 1.9–2.6) (Fig. 5a). Estimated t_½el_ was significantly higher in children treated with DHP compared to AA and AL [2.8 days (95% CI 2.1–3.5, *n* = 146) versus 1.6 days (95% CI 1.3–2, *n* = 138) versus 2.2 days (95% CI 1.7–2.8, *n* = 148), respectively *P* = 0.01] but it was similar between AA and AL (*P* = 0.27). Estimated t_½el_ was also significantly higher in children who were non-anaemic compared to anaemic children at presentation [2.7 days (95% CI 2.2–3.1, *n* = 318) versus 1.1 days (95% CI 0.9–1.2, *n* = 114) *P* < 0.0001] (Fig. 5b).

**Table 6:** Demographic and clinical characteristics of <5 years old children with uncomplicated falciparum malaria enrolled in study of the kinetics of the disposition of drug-attributable falls in haematocrit following artemisinin-based combination treatments

	Treatment				*P* value
	AA (*n* = 138)	AL (*n* = 148)	DHP (*n* = 146)	ALL (*n* = 432)	
Male/Female	78/60	74/74	82/64	234/198	0.45
Age (month)					
Mean (95% CI)	38.4 (35.6–41.2)	41 (38.5–43.6)	36.5 (33.6–39.2)	38.6 (37.1–40.2)	0.06
Age ≤ 24 [%]	36 [26]	27 [18]	53 [36]	116 [27]	0.002
Duration of illness (day)					
Mean (95% CI)	3.6 (3.1–4.1)	3.6 (3.1–4.1)	3.4 (2.9–3.9)	3.5 (3.2–3.8)	0.79
Temperature (°C)					
Mean (95% CI)	37.9 (37.7–38.1)	37.9 (37.5–38.2)	37.8 (37.4–38.2)	37.9 (37.6–38.1)	0.76
No. with temperature					
> 37.4 °C [%]	94 [68]	104 [70]	95 [65]	293 [68]	0.63
> 40 °C [%]	9 [6.5]	4 [3]	7 [5]	20 [5]	0.29
Haematocrit (%)					
Mean (95% CI)	31.8 (31–32.6)	31.9 (31.2–32.7)	32.3 (31.5–33)	32 (31.6–33.4)	0.67
No. with haematocrit <30% [%]	44 [32]	40 [27]	34 [23]	114 [26]	0.54
Parasitaemia (μL^−1^)					
Geometric mean	17,737	22,202	20,767	20,210	0.36
Range	2000–200,000	2004–200,000	2003–200,000	2000–200,000	
No with parasitaemia					
> 100,000 [%]	90 [65]	102 [69]	99 [68]	291 [67]	0.84
Gametocyte carriage [%]	12 [8.7]	7 [4.7]	11 [7.5]	30 [6.9]	0.38
Fever clearance time (day)					
Mean (95% CI)	1.1 (1.1–1.2)	1.3 (1.2–1.4)	1.2 (1.1–1.3)	1.2 (1.2–1.3)	0.07
Parasite positivity					
On day 1 [%]	75 [54]	98 [66]	81 [55]	254 [59]	0.07
On day 3 [%]	2 [1.4]	3 [2]	2 [1.4]	9 [2.1]	0.66
Parasite clearance time (day)					
Mean (95% CI)	1.8 (1.6–1.9)	1.9 (1.8–2)	1.7 (1.6–1.8)	1.8 (1.7–1.9)	0.054

*AA* artesunate-amodiaquine, *AL* artemether-lumefantrine, *DHP* dihydroartemisinin-piperaquine, *CI* confidence interval

Overall, estimated mean estimated AUC_(DAFH)_ was 56.3%.day (95% CI 48.5–64.2). Estimated mean AUC_DAFH_ was significantly higher in children treated with DHP compared to AA and AL [67%.day (95% CI 57.4–82.6, *n* = 146) versus 41.7%.day (95% CI 32.5–50.8, *n* = 138) versus 59.5%.day (95% CI 44.7–74.3, *n* = 148), respectively *P* = 0.03] but it was similar between AA and AL (*P* = 0.16). Estimated mean AUC_DAFH_ was also significantly higher in children who were non-anaemic compared to anaemic children at presentation [66.1%.day (95% CI 57–75.2, *n* = 317) versus 29.3%.day (95% CI 14.5–44.1, *n* = 114) *P* < 0.0001].

### Relationship between area under curve of the plot of deficit in drug-attributable falls in haematocrit and value of drug-attributable falls in haematocrit

**Fig. 6:**
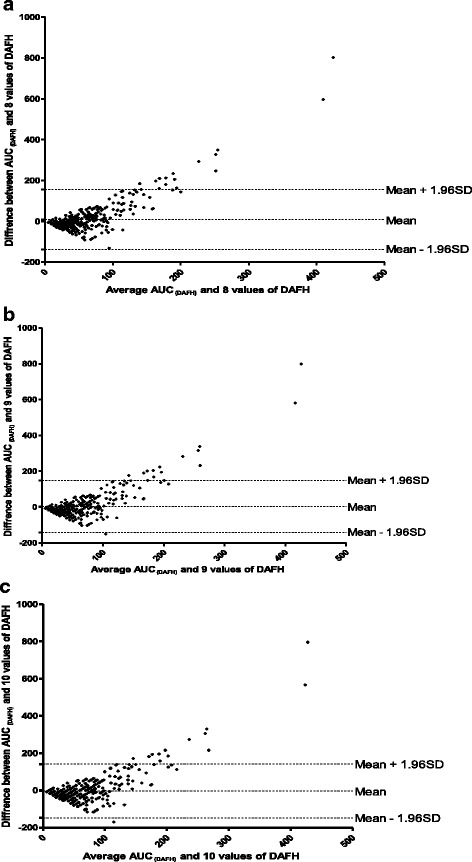
Bland-Altman plots of area under curve of drug-attributable fall in haematocrit (AUC_(DAFH)_) and multiples [8 (**a**), 9 (**b**) and 10 (**c**)] of values of drug-attributable falls in haematocrit. Biases were 9.18, 3.27 and −2.63 for plots A, B and C; *P* = 0.01, 0.36 and 0.46, respectively. The mean values ±1.96 standard deviation (SD) of the differences are shown

The relationship between AUC_(DAFH)_ and the value of DAFH was evaluated in 432 children. The mean of the ratio of AUC_(DAFH)_: value of DAFH was 8.8 (95% CI 7.4–10.3). There was a significantly positive correlation between AUC_(DAFH)_ and value of DAFH (*r* = 0.47, *P* < 0.0001). Bland-Altman plots of AUC_(DAFH)_ and multiples of values of DAFH are shown in Fig. 6. The limits of agreement between AUC_(DAFH)_ and 9 or 10 multiples of value of DAFH were narrow. At 9 and 10 multiples of value of DAFH, the limits of agreement were −142.1 – 148.6% and −147.4 – 142.1%, respectively. The bias at multiples of 9 or 10 value of DAFH was statistically insignificant (*P* = 0.36 and 0.46, respectively) indicating that AUC_(DAFH)_ and 9 or 10 multiples of value of DAFH can be used interchangeably in the same patient. However, there was a statistically significant bias at 8 multiples of value of DAFH (*P* = 0.01).

## Discussion

In this study, we evaluated prospectively in young malarious children, the factors contributing to: anaemia at presentation, anaemia one week after starting treatment in children who were not anaemic at presentation, the burden of anaemia imposed by a single episode of malaria, the falls and the duration of falls in haematocrit attributable to three ACTs. Our results showed: anaemia is common at presentation and in the one week following initiation of treatment; the burden of anaemia after a single episode of infection is high; and drug related falls in haematocrit are significantly more common and more severe in children who were not anaemic at presentation compared with those who were anaemic at presentation.

Compared with factors contributing to anaemia during the early period of adoption of ACTs as first-line antimalarials in the study areas [8], the factors contributing to anaemia ten years after adoption as first-line treatments appeared to have been considerably modified as shown in the results of the present study. The significant modifications include: reduction in age band susceptible to malarial anaemia at presentation from children 6–59 months to children 6–15 months and relatively low gametocyte carriage at presentation or following treatment. However, the prevalence of anaemia at presentation and the burden of anaemia following a single episode of infection appeared to have been little affected. The low gametocyte carriage ten years following adoption as first-line antimalarials can be attributable to rapid clearance of asexual parasitaemia [17, 23, 30, 31] and alteration in gametocyte sex ratios [32, 33] which can produce a female bias sex ratio that is less infective to mosquitoes [34]. It has also been postulated that ACTs can mobilize immature gametocytes from bone marrow to peripheral blood where they can be rapidly eliminated by components of ACTs [30, 33]. The latter can also prevent development of immature gametocytes which are non-infective to mosquitoes to mature gametocyte which are infective to mosquitoes [30, 33]. This is particularly important because all components of ACTs can kill immature gametocytes. It is not apparent from the results of the present study why a low asexual parasitaemia is a predictor of anaemia at presentation. This finding is paradoxical.

Progression to anaemia within one week of treatment initiation in a half of the children who were not anaemic at presentation can be clearly attributable to relatively high DAFH values with all three treatments. These children differ significantly from anaemic children at presentation in whom DAFH values were relatively low and who did not have a worsening of their anaemia in the one week following treatment initiation (Fig. 5). This finding in anaemic children suggests considerable degree of “haematocrit conservation” [31, 35]. Taking together, this would suggest the three ACTs may prevent further falls in haematocrit in anaemic children at presentation and in half of non-anaemic children at presentation.

The burden of anaemia imposed by a single episode of infection was relatively high and it occurred in two-third of the young malarious children. In these children, repeated attacks of acute infections within a short time frame can be expected to lead to development of anaemia [16]. Expectedly, anaemia and gametocytaemia at presentation predicted MAFH >4%. Indeed the risk of MAFH >4% was approximately nine and a half fold higher in anaemic compared to non-anaemic children at presentation (Table 4). Taking together with factors predicting anaemia at presentation, it would appear that by reducing the age band susceptible to malaria at presentation from 6 to 59 months to 6–15 months, ACTs have, during the ten years of adoption as first-line treatments, not reduced the burden of anaemia imposed by a single episode of an apparently uncomplicated infection. We have no ready explanation for a presenting parasitaemia ≤50,000 μL^−1^ being a predictor of MAFH in excess of 4%.

The early-appearing anaemia in 50% of non-anaemic children was clearly attributable to DAFH in excess of 4% (see above, paragraph 3). Therefore, all of the predictors of DAFH in excess of 4% were not unexpected (Table 5). However, there is no ready explanation from the results presented for temperature in excess of 37.9°C being a predictor of DAFH in excess of 4%. In this context, there is need to justify the use of DAFH >4% as cut off for predictors of DAFH. Firstly, this cut off has been used in a previous study [16]. Second, a ≥ 5% (5 units) fall in haematocrit from baseline is unlikely to be a random effect and likely to represent a significant fall from baseline in a manner similar to using 95% confidence interval in a two-way analysis of variance [30].

An important question arising from the present study is whether there are differences in the recovery from anaemia attributable to infections (pre-treatment anaemia) and the anaemia which follows treatment of the infections in patients who were not previously anaemic at presentation (early-appearing anaemia). Overall, it would appear there are significant differences as evidenced by the significantly longer anaemia recovery time in children with anaemia primarily due to the infections compared to the anaemia following treatment of the infections. Additionally, pre-treatment anaemia is primarily due to interaction(s) between host and the parasites while the anaemia following treatment is due to interaction(s) between host, parasite and the drug. A striking feature of all non-anaemic children pre-treatment who progressed to early-appearing anaemia were: most had pre-treatment haematocrit close to the lower threshold of normal and the critical falls in haematocrit at which all progressed to anemia was a drug-attributable fall in excess of 10% (Fig. 4). Perhaps determining threshold for progression of all those developing early appearing anaemia should form routine part of the clinical evaluation of drug-attributable falls in haematocrit following different drug regimens. The pre-treatment haematocrit threshold in the cohort of children with normal haematocrit at presentation appears to be a haematocrit of 40%.

The ratio of the estimated AUC_(DAFH)_ to value of DAFH was approximately nine folds. This ratio represents the relationship of a pharmacokinetic estimate to a pharmacodynamic estimate - two methods of measuring DAFH in the same patients employed in the present study. An intriguing feature of the ratio is: it is somewhat similar to the number of half-times required for >99.9% completion of a pharmacokinetic process in a rudimentary one-compartment pharmacokinetic model. A somewhat more intriguing finding is their insignificant agreement when 9 or 10 multiples of value of DAFH and AUC_(DAFH)_ were subjected to Bland-Altman analyses. The analyses indicate both measurements can be used interchangeably in the same patients despite the difference in their units of measurement. The difference in units of measurement would suggest some further application of Bland-Altman analysis when applied to establishing agreement between pharmacokinetic and pharmacodynamic processes in clinical drug studies.

The main limitation of the present study is not quantifying once-infected red blood cells. Quantification of once-infected red blood cells would have enabled the study to distinguish between non-anaemic children at presentation who progressed to early-appearing anaemia from those who did not. Another limitation is that the study did not evaluate the contribution of the background causes of anaemia, to the factors contributing to anaemia pre- and post-treatment initiation.

## Conclusions

In conclusion, after ten years of adopting of ACTs as first-line antimalarials in Nigeria, anaemia is common pre-and early post-treatment, anaemia burden of a single infection is relatively high, and drug-attributable falls in haematocrit >4% is common and significantly lower in anaemic compared to non-anaemic malarious children.

## Abbreviations

%: Percent
^o^C: Degree celsius
AA: Artesunate-amodiaquine
ACTs: Artemisinin-based combination treatments
AL: Artemether-lumefantrine
AOR: Adjusted odds ratio
AUC: Area under curve
AUC_(DAFH)_: Area under curve of drug-attributable falls in haematocrit versus time
CI: Confidence interval
d: Day
DAFH: Drug-attributable falls in haematocrit
DHP: Dihydroartemisinin-piperaquine
EAA: Early-appearing anaemia
g: Gram
kg: Kilogram
L: Litre
M/F: Male/female
MAFH: Malaria-attributable fall in haematocrit
mg: Milligram
OR: Odds ratio
t_1/2_: Half-time
TMAFH: Total malaria-attributable fall in haematocrit
μL: Microliter
